# Bacterial Community in Water and Air of Two Sub-Alpine Lakes in Taiwan

**DOI:** 10.1264/jsme2.ME17148

**Published:** 2018-04-21

**Authors:** Kshitij Tandon, Shan-Hua Yang, Min-Tao Wan, Chia-Chin Yang, Bayanmunkh Baatar, Chih-Yu Chiu, Jeng-Wei Tsai, Wen-Cheng Liu, Sen-Lin Tang

**Affiliations:** 1 Biodiversity Research Center, Academia Sinica Taipei 11529 Taiwan; 2 Bioinformatics Program, Institute of Information Science, Taiwan International Graduate Program, Academia Sinica Taipei 11529 Taiwan; 3 Institute of Bioinformatics and Structural Biology, National Tsing Hua University Hsinchu 30013 Taiwan; 4 EcoHealth Microbiology Laboratory, WanYu Co., Ltd. Chiayi 600 Taiwan; 5 China Medical University, Department of Biological Science and Technology Taichung Taiwan; 6 Department of Civil and Disaster Prevention Engineering, National United University Miao-Li 36003 Taiwan

**Keywords:** airborne, sub-alpine montane lakes, bacterial community, *Parcubacteria*

## Abstract

Very few studies have attempted to profile the microbial communities in the air above freshwater bodies, such as lakes, even though freshwater sources are an important part of aquatic ecosystems and airborne bacteria are the most dispersible microorganisms on earth. In the present study, we investigated microbial communities in the waters of two high mountain sub-alpine montane lakes—located 21 km apart and with disparate trophic characteristics—and the air above them. Although bacteria in the lakes had locational differences, their community compositions remained constant over time. However, airborne bacterial communities were diverse and displayed spatial and temporal variance. *Proteobacteria*, *Actinobacteria*, *Bacteroidetes*, and *Cyanobacteria* were dominant in both lakes, with different relative abundances between lakes, and *Parcubacteria* (OD1) was dominant in air samples for all sampling times, except two. We also identified certain shared taxa between lake water and the air above it. The results obtained on these communities in the present study provide putative candidates to study how airborne communities shape lake water bacterial compositions and vice versa.

Microorganisms are ubiquitous and govern biogeochemical cycles on a global scale (19). They have been found in almost every niche on earth, including soil (21, 45), the ocean (50), and the human gut (41). Bacteria are the most important components of microbial communities in aquatic ecosystems and are responsible for the mineralization of organic matter and nutrient recycling processes (3). However, the challenges and difficulties associated with cultivating most bacterial species resulted in the factors driving bacterial community compositions remaining elusive, at least until the last decade (36). Advances in sequencing technologies and the introduction of new and improved metagenomics tools have allowed microbial ecologists to intensively investigate bacterial community compositions and factors driving diversity, in relation to both the function and ecology of aquatic ecosystems.

Microbiological studies have focused on the bacterial composition of lakes (29, 34, 39, 55), in addition to other freshwater systems, such as rivers (32) and ponds (13). A recent review by Newton *et al.* (38) provided detailed information on the bacterial communities of the epilimnetic waters of lakes worldwide, with *Proteobacteria*, *Actinobacteria*, *Bacteroidetes*, *Cyanobacteria*, and *Verrucomicrobia* being the dominant phyla. Moreover, we hypothesize that some of these bacteria may also be present in the air above these freshwater bodies.

Airborne bacteria have been studied in recent years in order to ascertain their role as bioaerosols (2). They have been associated with ice-nucleation in clouds (6, 14, 36) and have also been linked to hypersensitive diseases (*i.e.*, humidified fever and asthma) based on epidemiological studies (20). Dust storms carry bacteria into new and remote environments (5). Airborne bacteria have been shown to travel long distances because their small size allows them to adhere to dust particles; they subsequently change microbial community compositions in the environments they travel to (7). Backward trajectory models have been used to study the movement of these airborne bacteria (4, 18) and elucidate how these migrating bacteria alter dynamics in their new habitats.

Recent studies have attempted to investigate bacterial air and lake communities in various geographical landscapes using high-throughput approaches (12) and microcosm experiments (28); these studies have shown that bacterial air and lake communities are both variable and dynamic (10, 11). Moreover, air bacteria are composed of a wide range of taxa from different sources that may sustain a wide range of environmental conditions (28) and consequently survive migration to novel environments (11); high mountain lakes are one of these environments. It currently remains unclear whether the bacterial compositions of these lakes affect air passing over them or vice versa. Therefore, we are interested in investigating the bacterial communities of lake water and the air above it and using this information to identify candidates for bacterial taxa that may colonize both habitats.

Tsuei-Feng Lake (TFL) and Yuan-Yang Lake (YYL) are the only freshwater sub-alpine lakes in north-central Taiwan. They share similar geographical characteristics—latitude, altitude (1,840 m of TFL and 1,670 m of YYL), and limited anthropogenic activity—and are both in protected areas. In addition, rainfall is the only source of water for these lakes. They have different nutrient levels because TFL is oligotrophic (54) and YYL is mesotrophic (52). Based on their shared geographical characteristics and close proximity (21 km), we hypothesized that they have similar air masses affecting them. Hence, they are ideal for investigating the dynamics of bacterial community compositions in and directly above lakes.

In the present study, we used a 16S rDNA Illumina sequencing approach to characterize the bacterial communities in and directly above the two lakes at four time points between January and February, 2015. Our goal was to investigate bacterial compositions and attempt to decipher the influence (if any) of airborne bacteria on lake water communities and vice versa.

## Materials and Methods

### Sampling sites

Yuan-Yang Lake (24° 34′ 33.6″ N, 121° 24′ 7.2″ E) is located 1,670 m above sea level, while Tsuei-Feng Lake (24° 30′ 52.5″ N, 121° 36′ 24.8″ E) is 1,840 m above sea level. The two lakes are approximately 21 km apart, and both are sub-alpine montane lakes in Taiwan’s Hsinchu and Yilan counties, respectively.

### Collecting air samples

Samples of air above the surfaces of these lakes were collected between January and February 2015. In order to obtain air samples, equipment was fabricated with a motor (Model PZX512BL; Ever Motor Guanlian Corp., Taiwan) connected to a portable battery (YUASA Battery, Taipei, Taiwan) and cellulose acetate membranes (pore size 0.22 μm, diameter 47 mm, Advantec, Tokyo, Japan). Air samples were collected for 1 h during each sampling time on the shores of the lakes at a height of 1 m. The flow rate was measured using the Dwyer RMA-21-SSV Flow meter (Global Test Supply, NC, USA) and was 2 L min^−1^. After sampling, the cellulose acetate membrane was carefully transferred to a 15-mL polystyrene tube and placed at −20°C until DNA extraction in the laboratory. There was no rainfall at any of the sampling times (±3 h), even though humidity was high on certain days (Table S1).

### Collecting lake samples

Surface water (depth of 1 m) samples were collected from a boat at the deepest points of the lakes at the same time as air samples. Two liters of lake water was initially filtered using a piece of gauze (to remove large debris such as leaves), followed by an 11-μm filter to remove sand, dust particles, and planktonic organisms, and 200 mL of water was then filtered with a 0.22-μm (diameter of 47 mm, Advantec) filter. Time replicates (*n*=3) were collected at each sampling point. Samples were kept at −20°C before DNA extraction in the laboratory.

### Data availability and sample representation

Raw sequenced reads were submitted to NCBI under Bioproject PRJNA393066. The representation of samples and environment parameters measured during sampling time points are shown (Table S1). Samples are hereafter represented as YA (Yuan-Yang Air), TA (Tsuei-Feng Air), YS (Yuan-Yang surface water), and TS (Tsuei-Feng surface water). The suffix (1, 2, 3, and 4) indicates the sampling times.

### DNA extraction, PCR, and sequencing

Genomic DNA was extracted from air and surface water samples using the UltraClean Soil DNA Kit (MioBio, Solana Beach, CA, USA) following the manufacturer’s protocol. The quality and quantity of extracted nucleic acids were measured using a Scandrop spectrophotometer (Thermo Scientific, Vantaa, Finland).

16S rRNA gene amplification was performed using the bacterial universal primers 968F (5′-AACGCGAAGAACCTTAC-3′) (8) and 1391R (5′-ACGGGCGGTGWGTRC-3′) for the V6–V8 hypervariable region (31).

The reaction mixture contained 1 μL of 5 U TaKaRa *Ex Taq* HS (Takara Bio, Otsu, Japan), 5 μL of 10× *Ex Taq* buffer, 4 μL of 2.5 mM deoxynucleotide triphosphate mixture, 1 μL of each primer (10 μM), and 1–5 μL (10–20 ng) of template DNA in a volume of 50 μL. PCR was performed under the following conditions: 94°C for 5 min, 30 cycles of 94°C for 30 s, 52°C for 20 s, and 72°C for 45 s, with a final extension of 72°C for 10 min.

The PCR amplicons of the bacterial 16S rRNA gene’s V6–V8 region were verified by DNA agarose gel electrophoresis with a 1.5% agarose gel and 1× TAE buffer. The expected DNA band (~320 bp) was cut from the gel, DNA was recovered with the QIAEX II Agarose Gel Extraction Kit (QIAGEN, Hilden, Germany), and quality was verified with the Scandrop spectrophotometer (Thermo Scientific).

DNA-tagging PCR was used to tag each PCR product of the bacterial 16S rRNA gene’s V6–V8 region (8). The tag primer was designed with four overhanging nucleotides; this arrangement ensured 256 distinct tags—at the 5′ end of the 968F and 1391R primers—for bacterial DNA. The tagging PCR conditions consisted of an initial step of 94°C for 3 min, 5 cycles at 94°C for 20 s, 60°C for 15 s, 72°C for 20 s, and a final step of 72°C for 2 min.

Illumina sequencing (Miseq, Yourgene Biosciences, Taiwan) was performed on pooled 40-ng lots of uniquely marked samples (47 in total). A TruSeq DNA-PCR Free library was prepared for 2×300 bp paired-end reads by Yourgene Biosciences. Raw reads were sorted and primers removed before further analyses.

### Data analysis

Reads obtained from Illumina sequencing were quality filtered using Mothur v1.38.1 (46) on a per sample basis. Quality-filtered reads (minimum length 350 bp and maximum length 450 bp, average quality score >27) were retained for further analyses. Reads containing any ambiguous base or homopolymer >8 bp were removed. Chimeric reads were identified and removed by UCHIME with USEARCH v8 (parameters: reference mode, rdp gold database, and mindiv of 3) (16). Qualified sequences were retained for subsequent analyses.

Operational Taxonomic Units (OTUs) were generated from filtered and non-chimeric reads using the UPARSE pipeline (17) with close reference (97% identity). The OTU classification was performed on a per sample basis using the SILVA v128 database (42, 60) with a pseudo-bootstrap cut-off of 80%.

Air and surface water samples were rarefied separately (based on minimum read counts) due to large variations in their sequence counts. Air samples had 140,235 sequences ranging between 3,572 and 10,128. Surface water samples had 605,007 sequences that ranged between 8,375 and 51,720. Air samples were rarefied with 3,572 sequences and surface water samples with 8,375; rarefaction was performed in Mothur. Diversity measures (Richness, Good’s coverage, chao1, Shannon, Simpson, Ace, Simpson’s evenness, and Shannon’s evenness) were calculated with 1000 iterations on rarefied data with Mothur.

### Clustering analysis and shared bacterial taxa identification

The relative abundance (RA) of OTUs was calculated on unrarefied data using an in-house R script. A matrix of the bacterial RA of OTUs at the genus level was used to calculate the Bray-Curtis distance between each sample (air and surface water combined). Clustering with the group average method was performed at the phylum and class levels to reveal differences in taxa between air and surface water samples from two sampling sites via Primer6 (PRIMER-E, Lutton, Lvybridge, UK) (9). A non-metric multidimensional scaling (nMDS) analysis was performed in order to elucidate the relationship between bacterial communities in different habitats (and locations) using Primer6.

Shared OTUs (unrarefied data) among the four habitats were identified in all possible combinations and a Venn diagram was plotted using Venny 2.1. Shared OTUs were classified into four groups: Group A, present only in the air; Group B, present only in the lake; Group C, present in lake and air samples; Group D, present in all four habitats. Taxonomic distributions for all groups were analyzed and pie charts were prepared of distribution at the phylum level.

Since Group D OTUs were found in all four habitats, a Blastn (1) analysis of all 18 OTUs was performed and each sample’s annotation and potential source were also identified. We further calculated the contribution ratio of these OTUs with the equation below.

% contribution of OTU (in air or water)=(OTU abundance/Σ OTU abundance)*100

### Phylogenetic analysis

BLAST against a non-redundant nucleotide database (last accessed in July, 2016) was performed and hits were filtered based on a percent identity ≥90% and e-value <1e–05, with a maximum of five hits being obtained using the max_target_seqs option of standalone BLAST. FASTA sequences were extracted from the NCBI nucleotide database based on the accession IDs of the hits. Multiple sequence alignment was performed using MUSCLE (15) in MEGA7 (33) with default settings. Phylogeny was derived using the Maximum-Likelihood method with the Jukes-Cantor method, uniform rates, and complete deletion parameters with 1,000 bootstraps.

### Source estimation

The sequences of all 18 OTUs present in Group D were subjected to MetaMetaDB (59) for comprehensive source estimation (last accessed on November 8, 2017). MetaMetaDB performed BLAST against 1,241,213 representative 16S rRNA sequences and calculated the Microbial Habitability Index (MHI) in order to predict organisms’ native habitats. MHI scores were calculated as described by Yang *et al.* (59).

### Backward trajectory calculations

The backward trajectories of air masses were calculated using the Vertical Velocity National Oceanic and Atmospheric Administration (NOAA) Hybrid Single-Particle Lagrangian Integrated Trajectory (HYSPLIT) Model and the Global Data Assimilation System (GDAS1) meteorological database (43, 49). Trajectories were estimated using sequential runs (using “Start a new trajectory very” option: parameter 1 h), the starting point for backward tracing was the sampling location, and the duration was 72 h. This model has been adopted to estimate the sources of airborne microbes (48, 58).

## Results

### Microbial community diversity

In total, 690 OTUs were identified in surface water and air samples when analyzed together, with 662 OTUs in surface water and 68 OTUs in air samples when analyzed separately. When rarefied, there were 4 to 8 air sample OTUs and 76 to 182 surface water sample OTUs (Fig. S1 and S2). When diversity indices were calculated separately for air and surface water samples, no significant differences were observed in the Shannon, Simpson, or Evenness diversity (Shannon and Simpson) (Table S2) of air samples from the two lakes. Shannon diversity was significantly different with the factor of two lakes (ANOSIM R: 0.604, *P*<0.05), Simpson and Evenness diversities showed no significant differences with the factor of two lakes and the factor of four sampling times.

### Bacterial community composition

**Fig. 1:**
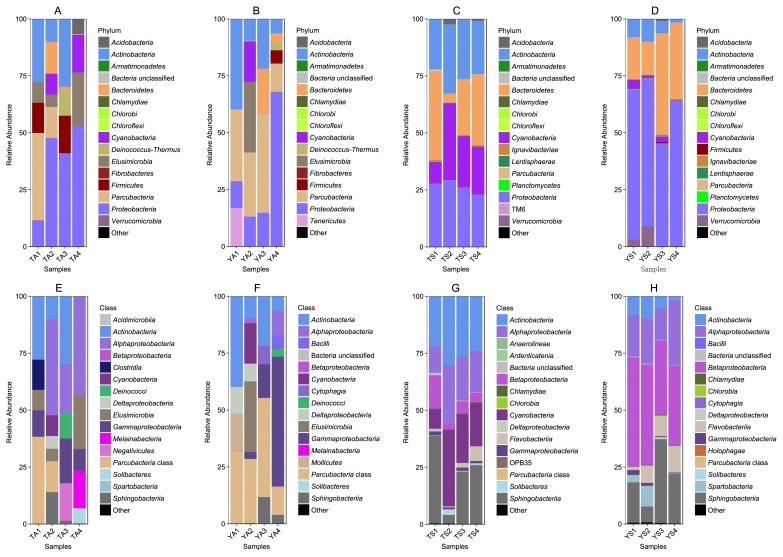
Relative abundances of bacterial phyla and classes in two lakes and air above. A–D are bacterial phyla and E–H are bacterial classes in the four habitats. A) and E) are bacterial compositions in the air at Tsuei-Feng Lake; B) and F) are bacterial compositions in the air at Yuan-Yang Lake; C) and G) are bacterial compositions in Tsuei-Feng Lake’s surface water; and D) and H) are bacterial compositions in Yuan-Yang Lake’s surface water. The x-axis indicates the four sampling times for each habitat. Colors indicate bacterial phyla and classes.

A difference was observed in bacterial community samples between the surface water of lakes and air (Fig. 1). In the air, bacterial phyla varied with the sampling time (Fig. 1A and B), whereas lakes had similar bacterial groups during sampling times (Fig. 1C and D). When the bacterial communities of the two surface water samples were compared, *Proteobacteria*, *Actinobacteria*, *Bacteroidetes*, and *Cyanobacteria* were dominant. However, RAs in these phyla were different between the two lakes. In terms of *Proteobacteria*, *Betaproteobacteria* was the dominant class in YS, whereas *Betaproteobacteria* had a lower abundance than *Alphaproteobacteria* in TS (Fig. 1G and H). *Actinobacteria* and *Cyanobacteria* had higher RAs in TS. *Verrucomicrobia* increased in abundance between the first two samplings in YS, but had a very low abundance in TS (Fig. 1D).

Bacterial communities in air samples varied between the locations and sampling times in each location. *Parcubacteria* (OD1) was dominant in two samples from TA (TA1 and TA2) and all samples from YA (Fig. 1E and F). In addition to OD1, *Actinobacteria* and *Proteobacteria* were also highly abundant.

**Fig. 2:**
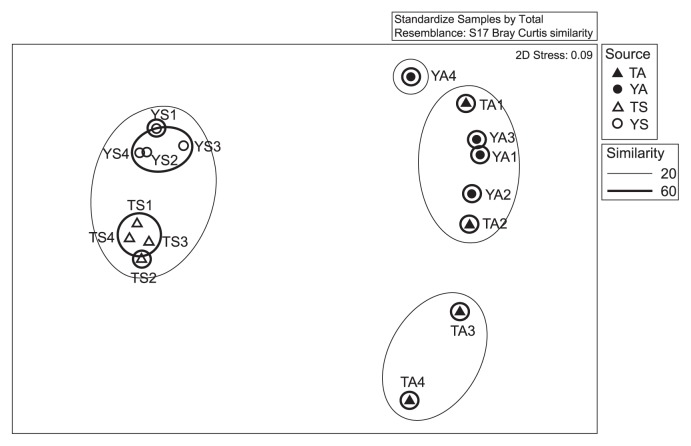
nMDS plot of bacterial genera in four habitats. Symbols indicate bacterial communities (genus level).

At the genus level, differences were observed in bacterial communities between lakes and air samples, corroborating results obtained at the phylum level (Fig. 2 and S3). In the nMDS analysis, bacterial genera in lake samples were separated by location, and intra-lake samples exhibited more than 60% similarity. However, air samples were not separated by locations at the genus level.

### Shared and unique OTUs

**Fig. 3:**
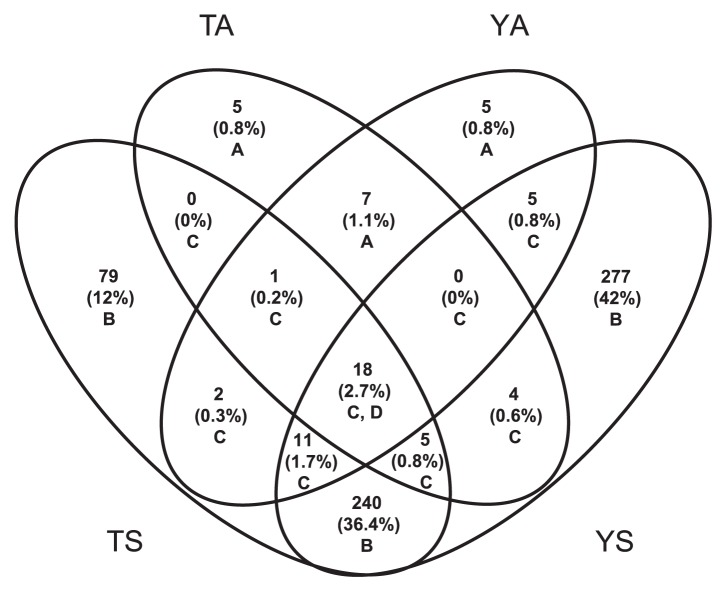
Venn diagram of shared OTUs and groups. A indicates the 17 bacterial OTUs obtained only from air above the two lakes; B indicates the 596 bacterial OTUs obtained only from the lakes; C indicates the 46 bacterial OTUs obtained in both lakes and air samples; and D indicates the 18 OTUs found in all four habitats.

YS had 560 OTUs, the highest among all four habitats, and TS had 356. YA had 49 OTUs, and 40 OTUs were identified from TA samples, with 18 OTUs shared among all four habitats, 7 OTUs shared by only air samples, and 240 OTUs by only lake samples. There were 5 and 5 unique OTUs for YA and TA samples, respectively, and 277 and 79 unique OTUs for YS and TS samples, respectively (Fig. 3).

Shared OTUs in various habitats were categorized into four groups (Fig. S4). *Alphaproteobacteria*, *Gammaproteobacteria*, *Actinobacteria*, and *Cyanobacteria* were dominant in Group A; bacterial OTUs present in only air samples. *Alphaproteobacteria* was dominant, followed by *Bacteroidetes*, in Group B; OTUs present only in lake water samples. In Group C, OTUs present in air and lake water samples, *Betaproteobacteria* had the highest RA, with *Parcubacteria* and *Elusimicrobia* also being present in this group. In Group D, which shared all 4 sample types (TA, YA, TS, and YS) and had 18 OTUs that overlapped in four habitats, *Parcubacteria* (OTU6) was the most abundant.

Although bacterial OTUs in Group D were identified in the lakes and air at the two locations, they were divided into two groups based on their RAs in lakes or air (Table 1). Overlapping OTUs, which had high RA in air, may have diverse environments in which they may live, including hot springs, soil, leaves, and animals. Moreover, OTUs abundant in lakes were also derived from various potential sources, such as hot springs, soil, fossils, ice, and marine and freshwater locations.

### Phylogeny of *Parcubacteria* (OD1)

**Fig. 4:**
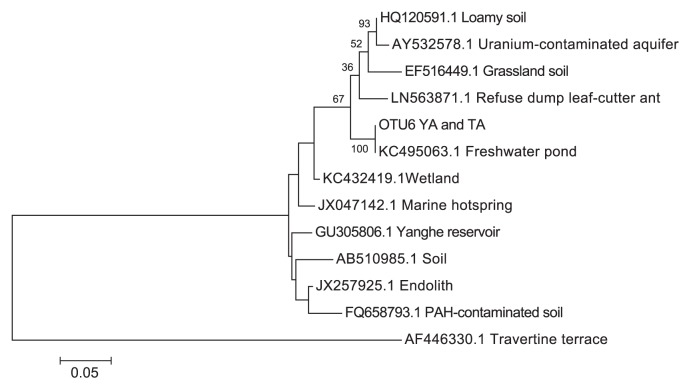
ML tree of OTU6 (OD1, *Parcubacteria*), generated with 1,000 bootstraps.

A phylogenetic analysis of OTU6 revealed that its closer relative (% identity ≥99%) was a clone sequence of *Candidatus* Sonnebornia vantaiensis (Fig. 4, Table 1), isolated from a freshwater pond in Shandong, China (GenBank: KC495063.1). An MHI analysis of all the sequences used in the phylogenetic analysis provided various potential sources.

### Source of air mass

Backward trajectory results indicated that air masses at each sampling time had different trajectories during the 72 h prior to reaching the two lakes. Air masses in the first and second sampling times at TFL (TA1 and TA2) and YYL (YA1 and YA2) mainly came from South-East Asia through a mix of oceanic and land travel (Fig. S5A and B). However, air masses from sampling times TA3 and TA4 along with YA3 and YA4 came from multiple locations, mainly through land travel (Fig. S5A and B). When trajectories were plotted every h, we observed that air masses for sample times TA3 and YA3 were mixed and traveled long distances and TA4 and YA4 mainly came from the Indian subcontinent (Fig. S5A and B). These results indicated that the two locations were subjected to both local and global masses.

## Discussion

The present study focused on bacterial community compositions in the water of and air above two sub-alpine lakes (TFL and YYL). Bacterial communities significantly differed between the water of the two lakes, possibly because the lakes had different trophic states (oligotrophic [54] and mesotrophic [52]). Moreover, there was a constant pattern of temporal variance. The two lakes were dominated by different bacterioplankton; TFL had more *Cyanobacteria* and members of the hgcI clade (*Actinobacteria*), whereas YYL was abundant in *Betaproteobacteria*, including the genera of *Albidiferax*, *Polynucleobacter*, and *Limnohabitans*. In addition, *Gammaproteobacteria*, *Sphingobacteria*, and a few other groups were detected.

The bacterial communities identified in the waters of sub-alpine lakes represented a typical freshwater community, similar to those reported in previous studies (22, 30, 35, 37). *Actinobacteria* were detected through time and space in both the lakes. The hgcI clade is common and abundant in freshwater habitats (56). A recent study using single-cell genomics showed that members of the hgcI clade metabolize carbohydrates and N-rich compounds and also utilize sunlight via actinorhodopsin, promoting anaplerotic carbon fixation (24). The widespread detection of *Actinobacteria* may be associated with a reduced cell wall type and cell size (26, 40, 51). *Betaproteobacteria* were the most abundant bacteria in epilimnetic lake water (38). The *Rhodoferax*, GSK, and *Limnohabitans* clades prefer algae-derived dissolved organic carbon, while *Polynucleobacter* from the BetII tribe prefers photo-oxidized products from humic lakes (27, 57). *Gammaproteobacteria* were not as abundant in the two lakes as that reported previously for freshwater lakes (3), even though culture studies suggested that bacteria in this group grow better in N- and P-enriched lakes (23, 47).

*Bacteroidetes* is the most dominant bacterial group in mesotrophic water bodies (29, 53). Among *Bacteroidetes*, the distribution of *Flavobacteriia* and *Sediminibacterium* in YYL waters and their absence from TFL waters suggest that they thrive better in this environment.

Our hypothesis that the same air mass affects both lakes was proven wrong because the backward trajectory analysis showed that the two lakes were subjected to air masses from different routes and sources (oceanic, terrestrial, and hybrid) at different sampling times. Terrestrial routes came from different regions of China, passing over the East China Sea (TA1, TA2); oceanic routes came from the Pacific Ocean (YA1, YA2); and hybrid routes combined the terrestrial and oceanic areas and traveled longer distances (TA3, TA4, YA3, YA4). These differences in routes may also affect airborne bacterial compositions above the two lakes. Airborne bacterial communities were similar during the first and second sampling, but varied significantly in the third and fourth sampling, suggesting a dynamic pattern influenced by air masses coming from different sources.

In terms of the bacterial composition in air, OTU6 was the most dominant taxon among *Parcubacteria* and had >99% similarity with *Candidatus* Sonnebornia yantaiensis from a freshwater pond in China (25). Since 16S rRNA (in the present study, V6–V8, 300–400 bp) NGS sequences are short, they may have multiple hits in the Non-Redundant database, and, hence, the specific source of bacteria may lead to ambiguous results. Therefore, in order to identify the ordinary habitat from which OTU6 originated, a MetaMetaDB analysis was performed, and soil (and sediment) was identified as the most significant habitat. Therefore, nearby terrestrial environments may have contributed to the airborne community. Moreover, identifying soil as the predominant habitat for other shared bacterial taxa also confirmed that air may help transport bacterial species from one environment to another, dispersing and depositing bacterial communities in different habitats.

Lake shores are potential sources for airborne bacteria near lakes and above a lake’s water. Furthermore, terrestrial environments play a vital role in contributing bacteria to air. The identification of shared bacterial OTUs between air and lake water may contribute to the identification of bacteria species with the ability to inhabit both environments. In the present study, the diverse habitats of shared bacterial species provided an example of the transport of bacteria with air as a medium. One caveat in identifying shared and unique OTUs based on unrarefied data is variations due to the sequencing depth. If we sequence at a higher depth, the count of OTUs shared between habitats may increase in number. Bacterial dispersal and consequent deposition have been reported as a major cause of shifts in bacterial communities during dust storms in Asia (7) and Europe (44). Even though the present study does not provide evidence for interactions between lake water and air, identifying certain bacteria in both environments provides putative candidates to study their influence on bacterial communities in lake water.

In conclusion, we observed variations in bacterial communities in air over time above both lakes and hypothesize that different trophic states account for these differences in the communities between the 2 lakes. We also identified certain bacterial species that have diverse original habitats and may have originated from soil on the shore or water from the lakes to air, as suggested by the MHI analyses. These are putative candidates, and long- and short-term interval observations may provide a deeper understanding of their roles in shaping the bacterial communities of lake water.

## Supplementary Material



## Figures and Tables

**Table 1:** Relative abundances (RA), annotation, putative sources, and MetaMetaDB source estimation of 18 OTUs shared in four habitats

OTU	RA in air	RA in lakes	Annotation	%identity	Source in the NCBI hit	MetaMetaDB habitat
Relative abundance higher in lakes						

OTU1	<0.01	10.92	*Pseudanabaena galeata*	99.51%	NA	Hydrocarbon, soil
OTU2	<0.01	11.95	Uncultured bacterium	97.40%	High mountain lake	Soil, ice, marine
OTU4	<0.01	9.71	*Polynucleobacter necessaries*	100%	River water	Ice, marine
OTU5	<0.01	8.57	Uncultured bacterium	100%	Lake water	Ice, phyllosphere, soil, fossil
OTU7	<0.01	3.96	Uncultured bacterium	99.02%	Snow worm	Ice, soil, hot spring, marine
OTU247	<0.01	0.28	*Betaproteobacteria*	99.75%	Acid-impacted lake	Ice, freshwater

Relative abundance higher in air						

OTU6	22.7	0.03	*Candidatus* Sonnebornia yantaiensis	100%	Freshwater pond	Soil
OTU13	10.63	<0.01	*Elusimicrobium* sp.	99.51%	Forest soil	Hot springs, soil
OTU17	9.75	0.07	Uncultured bacterium	100%	Anaerobic reactor	Human nasal pharyngeal
OTU40	3.33	<0.01	Uncultured bacterium	99.75%	Coral	Soil
OTU35	2.47	<0.01	Uncultured bacterium	99.75%	Contaminated sediment	Soil
OTU39	2.20	<0.01	*Deltaproteobacteria*	99.51%	Abdomen	Soil, gut
OTU46	1.95	0.04	*Mycobacterium chelonae*	100%	NA	Compost
OTU52	1.42	<0.01	Uncultured bacterium	98.78%	Soil	Soil, gut
OTU53	1.22	0.01	Uncultured bacterium	100%	Spiraling whitefly	Hydrocarbon, soil, gut, human gut, ant, bioreactor
OTU61	1.39	<0.01	*Vibrionimonas magnilacihabitans*	100%	Lake water	Soil
OTU70	0.76	0.03	Uncultured bacterium	99.02%	Gut	Soil
OTU218	0.13	0.02	*Sphingomona* sp.	100%	Cloud water	Phyllosphere
