# Supplementary material for: Bacterial Community in Water and Air of Two Sub-Alpine Lakes in Taiwan
Source: Microbes Environ. 2018 Apr 21;33(2):120–6. doi: 10.1264/jsme2.ME17148 (PMC6031399; doi:10.1264/jsme2.ME17148)
Supplement: Supplementary file 1 [file 33_120_s1.pdf]

Bacterial community in water and air of two sub-alpine lakes in Taiwan

Kshitij Tandon[1,2,3+], Shan-Hua Yang[1+], Min-Tao Wan[4], Chia-Chin Yang[1], Bayanmunkh Baatar[1],

Chih-Yu Chiu[1], Jeng-Wei Tsai[5], Wen-Cheng Liu[6], and Sen-Lin Tang[1*]

[1]Biodiversity Research Center, Academia Sinica, Taipei 11529, Taiwan

[2]Bioinformatics Program, Institute of Information Science, Taiwan International Graduate Program, Academia Sinica, Taipei 11529, Taiwan

[3]Institute of Bioinformatics and Structural Biology, National Tsing Hua University, Hsinchu 30013, Taiwan

[4]EcoHealth Microbiology Laboratory, WanYu Co., Ltd., Chiayi 600, Taiwan

[5]China Medical University, Department of Biological Science and Technology, Taichung, Taiwan

[6]Department of Civil and Disaster Prevention Engineering, National United University, Miao-Li 36003, Taiwan

[+]These authors contributed equally to this work and shared co-first authorship.

[*] Corresponding author email: sltang@gate.sinica.edu.tw

Running headline: Bacteria in air and lake

Table S1. Sampling information and sample identifier.

Location	Sampling time	Date (2015)	P[a]	AT[b]	ST[c]	H[d]	Air sample	Lake sample	Wind speed (m/sec)	Rainfall (+/- 3h)	Lake Depth (Maximum depth)
Tsuei-Feng Lake (TFL)	1	Jan. 14	817	16.5	10.9	78.9	TA1	TS1	0.746	NO	4.7 m
	2	Jan. 29	817	9.8	10.6	95.0	TA2	TS2	0.634	NO	
	3	Feb. 13	817	14.0	9.0	49.6	TA3	TS3	1.11	NO	
	4	Feb. 26	820	20.8	14.0	55.1	TA4	TS4	0.24	NO	
Yuan-Yang Lake (YYL)	1	Jan. 13	837	8.9	8.6	100.0	YA1	YS1	0.071	NO	4.5 m
	2	Jan. 28	833	5.5	8.7	100.0	YA2	YS2	0.089	NO	
	3	Feb. 12	836	9.3	9.2	87.2	YA3	YS3	0.966	NO	
	4	Feb. 25	840	17.1	14.5	84.0	YA4	YS4	0.555	NO	

[a]Air pressure (Bar)
[b]Air temperature (°C)
[c]Surface water temperature (°C)
[d]Humidity (%)

Table S2

Diversity Analyses of Samples (Rarefied data)

a) Lake Surface water Samples (YS, TS)

SAMPLES	OTUS	COVERAGE	CHAO1	SHANNON	SIMPSON	ACE	SHANNONEVEN	SIMPSONEVEN
TS1	128	0.994	167.61	2.37	0.184	201.7	0.4896	0.0431
TS2	68	0.663	103.25	1.40	0.151	152.9	0.3029	0.0288
TS3	92.33	0.997	112.43	2.39	0.169	112.2	0.5335	0.0665
TS4	98.66	0.996	134.65	2.33	0.158	164.3	0.5095	0.0646
YS1	181.33	0.990	307.25	2.89	0.127	432	0.5559	0.0435
YS2	182	0.990	297.21	2.62	0.149	363.3	0.5038	0.0403
YS3	76	0.998	86.03	2.48	0.164	89.64	0.5791	0.0841
YS4	132.33	0.993	234.84	2.82	0.089	311.27	0.5782	0.0848

b) Air Samples

SAMPLES	OTUS	COVERAGE	CHAO1	SHANNON	SIMPSON	ACE	SHANNONEVEN	SIMPSONEVEN
TA1	6.33	0.999	6.5	0.78	0.557	5.750	0.469	0.4882
TA2	8	0.669	6.5	1.27	0.306	6.183	0.620	0.7396
TA3	6.5	0.999	13.5	1.10	0.391	25.433	0.362	0.5333
TA4	5	0.999	13	1.28	0.335	22.169	0.451	0.6367
YA1	4	0.999	12.667	1.21	0.353	21.583	0.441	0.6208
YA2	8.33	0.999	6.666	1.39	0.276	2.333	0.545	0.7348
YA3	4.66	0.999	6	1.18	0.353	4.333	0.496	0.6622
YA4	7.33	0.999	6	0.99	0.425	4.148	0.415	0.5520

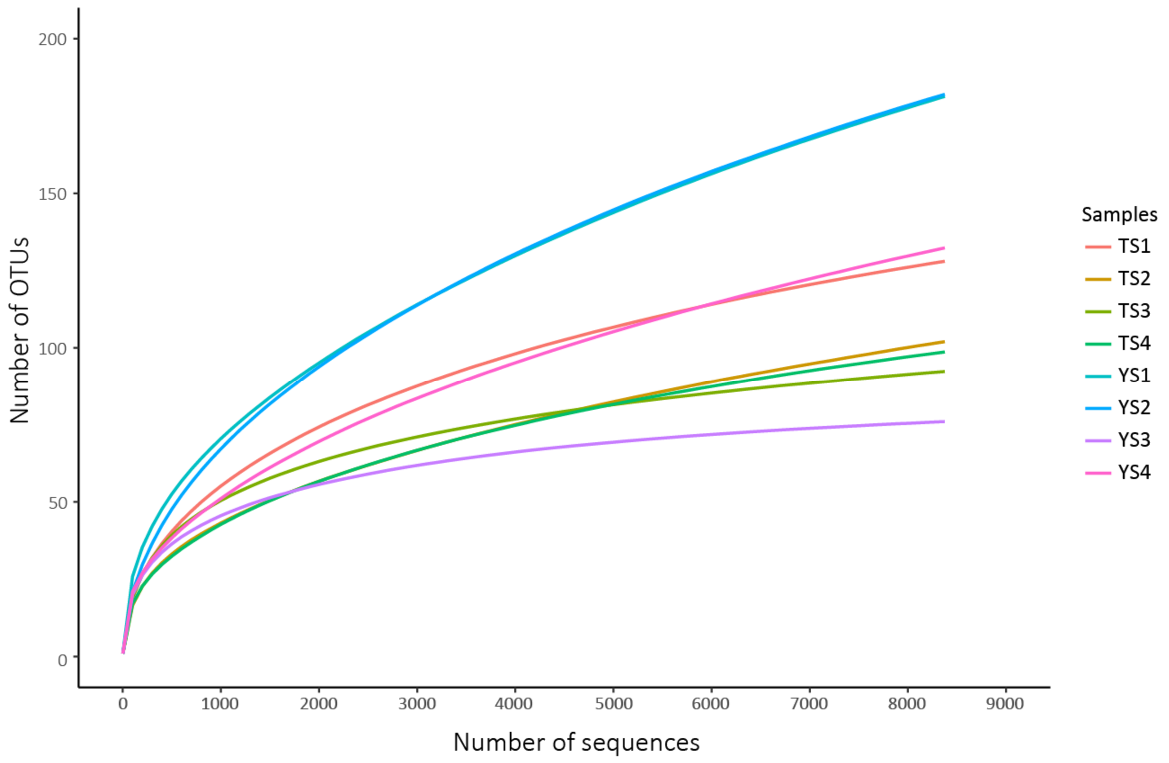

Figure S1. Rarefaction curves of bacterial OTUs in the surface water of the two lakes.

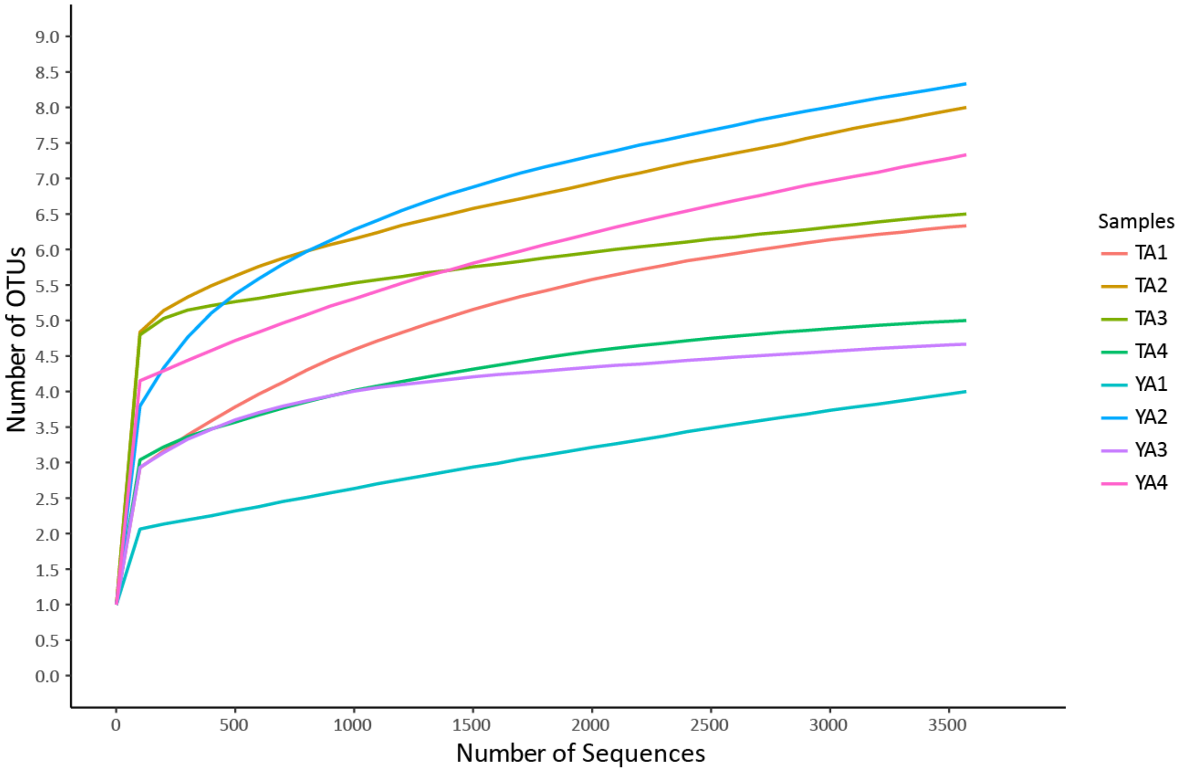

Figure S2. Rarefaction curves of bacterial OTUs in the air above the two lakes.

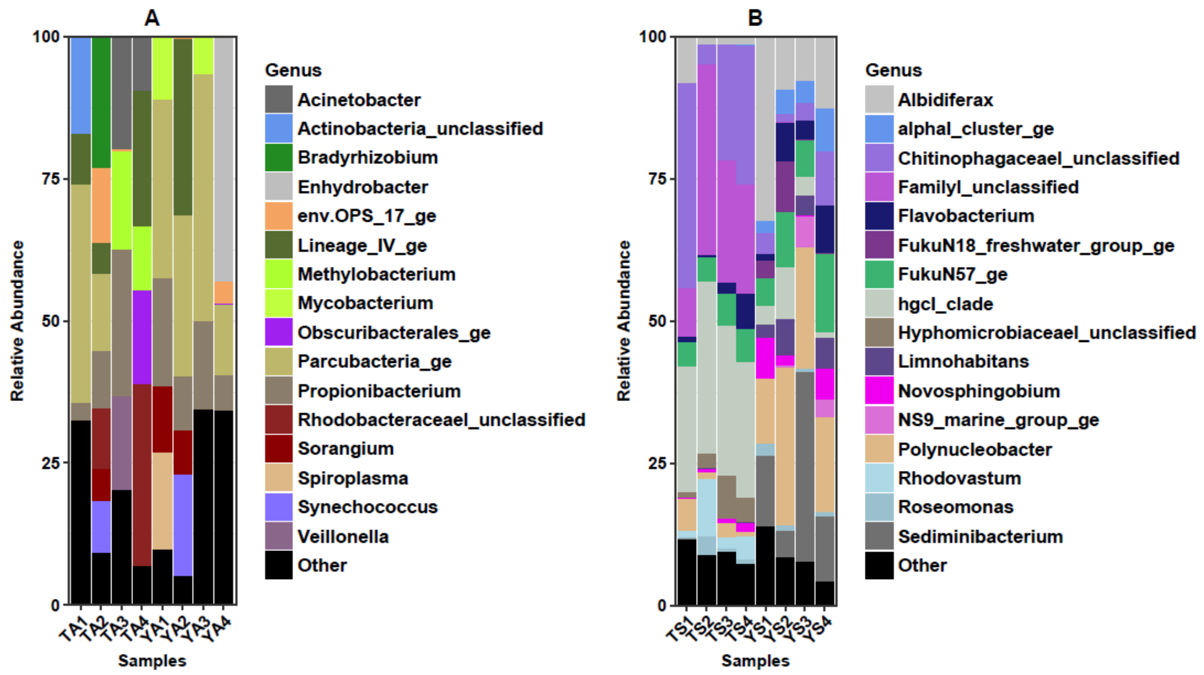

Fig S3. Bar plots for genus-level relative abundances for air and lake water samples. A) Relative abundances of the top 16 genera; *Parcubacteria* is the dominant group. B) Lake surface water samples relative abundances at the genus level. The two lakes had different compositions.

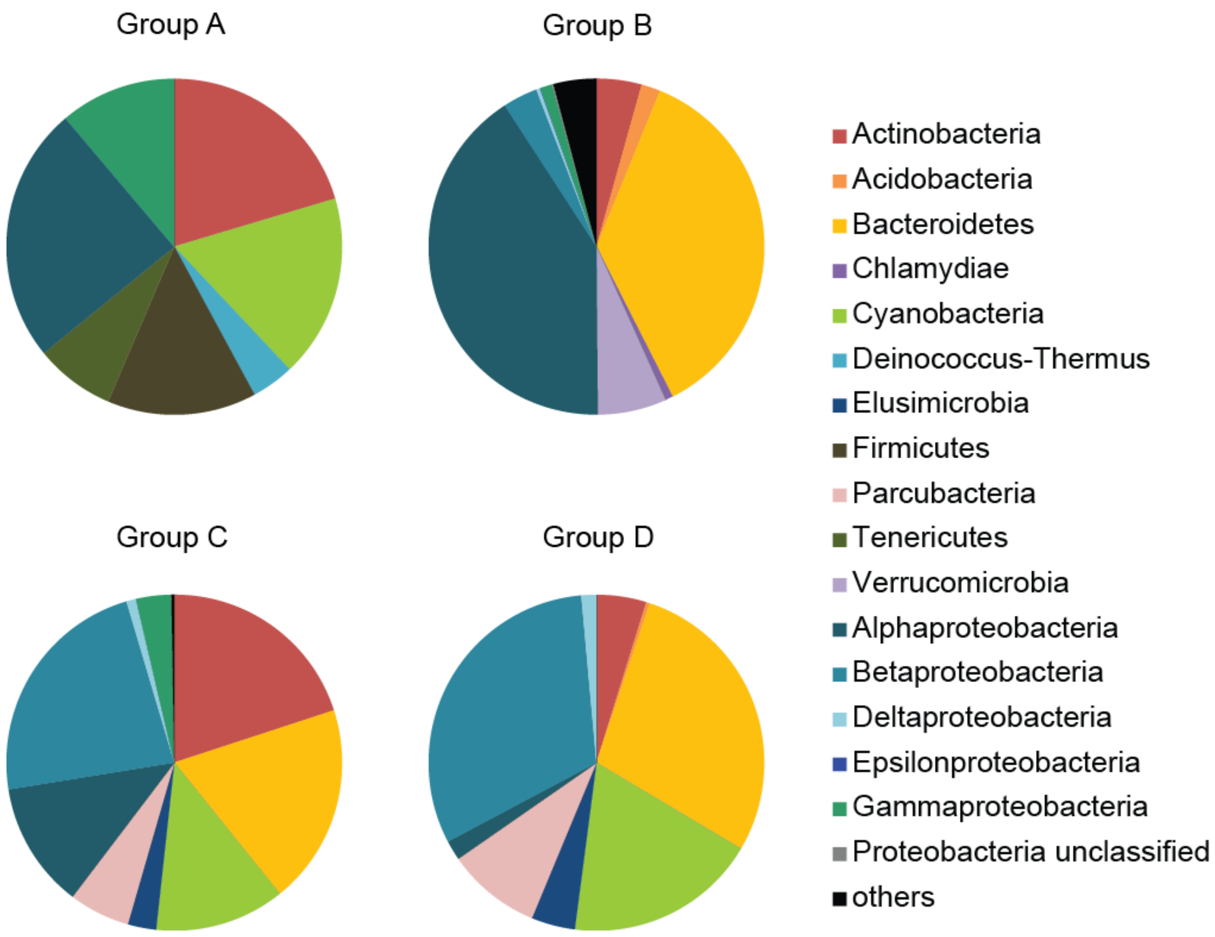

Figure S4. Pie charts of bacterial phylum and class levels (only for *Proteobacteria*) of the OTUs. Group A indicates 17 bacterial OTUs obtained only from the air above the two lakes (Group A in Figure 3); Group B indicates 596 bacterial OTUs obtained only from the lakes (Group B in Figure 3); Group C indicates 46 bacterial OTUs overlap in lakes and air samples (Group C in Figure 3); Group D indicates 18 bacterial OTUs that are shared among the four habitats (Group D in Figure 3). Colors represent different bacterial classes.

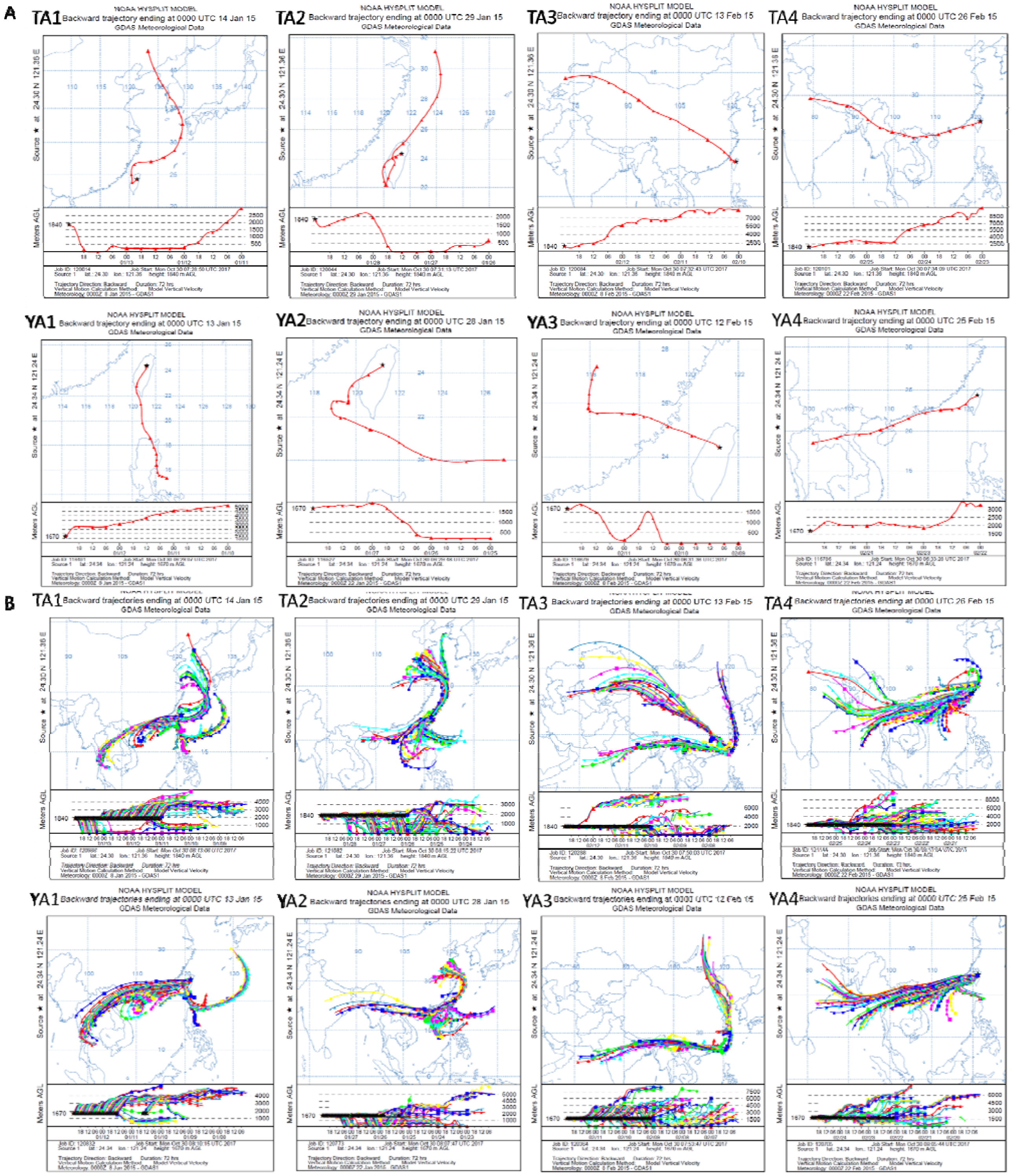

Figure S5. Backward trajectory simulations evaluated by HYSPLIT model for 72 h for each sampling time. TA1-TA4 are backward trajectories of sampling times 1-4 at Tsuei-Feng Lake; YA1-YA4 are backward trajectories of sampling times 1-4 at Yuan-Yang lake. A) Shows overall trajectory for 72 h and B)

shows air trajectories every 1 h for 72 h from the sampling day. Source of images: output of the public website service software HYSPLIT (http://ready.arl.noaa.gov/HYSPLIT.php).

Fasta Sequence OTU6

>OTU6

CAGGGCTAGAAACCAAGCTGCATCCAACCCGAAAGGGATGGAGCCTTCGAGGGT
GCTTGGCAGGTGATGCATGGCTGTCGTCAGTTCGTGGCTTGAGCTGTTCCCTTCA
GTGGGGTAACGAACGCAACCCTCGTTGTCTGTTAATTTTTTCAGGCGAGACTGCT
CCCTCACGGGAGAGGAAGGTGGGGATGACGCCAAGTCAGCATGTCCCTTGATGC
CCTGGGCTGCACACGTGATACAATGGGTGGTACAGAAGGACGCAATACGGTAAC
GTGGAGCAAATCCCAAAAACCACCCCCAGTACGGATTGAGGTCTGCAACCGACC
TCATGAAGCCGGAATCGCTAGTAATCGTGGGTCAGCCATACCACGGTGAATACG
TTCTCGAGTCTT
